# Characterization of Integrons and Resistance Genes in *Salmonella* Isolates from Farm Animals in Shandong Province, China

**DOI:** 10.3389/fmicb.2017.01300

**Published:** 2017-07-12

**Authors:** Xiaonan Zhao, Jie Yang, Baozhen Zhang, Shuhong Sun, Weishan Chang

**Affiliations:** ^1^College of Animal Science and Technology, Shandong Agricultural University Tai'an, China; ^2^Laboratory for Reproduction Health Institute of Biomedicine and Biotechnology Shenzhen Institutes of Advanced Technology, Chinese Academy of Sciences, Shenzhen University Town Shenzhen, China

**Keywords:** *Salmonella*, antimicrobial susceptibility, class 1 integron, antimicrobial resistance gene, MLST

## Abstract

A total of 154 non-duplicate *Salmonella* isolates were recovered from 1,105 rectal swabs collected from three large-scale chicken farms (78/325, 24.0%), three large-scale duck farms (56/600, 9.3%) and three large-scale pig farms (20/180, 11.1%) between April and July 2016. Seven serotypes were identified among the 154 isolates, with the most common serotype in chickens and ducks being *Salmonella* enteritidis and in pigs *Salmonella* typhimurium. Antimicrobial susceptibility testing revealed that high antimicrobial resistance rates were observed for tetracycline (72.0%) and ampicillin (69.4%) in all sources. Class 1 integrons were detected in 16.9% (26/154) of these isolates and contained gene cassettes *aadA2, aadA1, drfA1-aadA1, drfA12-aadA2*, and *drfA17-aadA5*. Three β-lactamase genes were detected among the 154 isolates, and most of the isolates carried *bla*_*TEM*−1_(55/154), followed by *bla*_*PSE*−1_(14/154) and *bla*_*CTX*−*M*−55_ (11/154). Three plasmid-mediated quinolone resistance genes were detected among the 154 isolates, and most of the isolates carried *qnrA* (113/154), followed by *qnrB* (99/154) and *qnrS* (10/154). *Fifty-four* isolates carried *floR* among the 154 isolates. Multilocus sequence typing (MLST) analysis showed that nine sequence types (STs) were identified; ST11 was the most frequent genotype in chickens and ducks, and ST19 was identified in pigs. Our findings indicated that *Salmonella* was widespread, and the overuse of antibiotics in animals should be reduced considerably in developing countries.

## Introduction

*Salmonella* is an important source of foodborne diseases that cause morbidity and mortality worldwide. Among 94 million cases of non-typhoid *Salmonella* infections, it was presumed that approximately 85% of the cases were induced by food origin *Salmonella* (Chiu et al., 2010). In China, *Salmonella* causes an estimated 22.2% of foodborne diseases (Wang et al., 2007). Many *Salmonella* serovars exist. More than 2,600 serovars are classified based on the reactivity of antisera to O and H antigens (Stevens et al., 2009), and the serovars from farms have a significant overlap with those causing illnesses in humans (Alcaine et al., 2006). Animals have been recognized as an important reservoir for *Salmonella*, and this pathogen can be transferred to humans via the food chain, posing a serious threat to human health (Vo et al., 2006).

The use of antimicrobials is important for the control and treatment of *Salmonella*. However, antimicrobial- and multidrug-resistant *Salmonella* strains have emerged, leading to treatment failure (Gong et al., 2013). The increasing prevalence of multidrug-resistance among *Salmonella*, not only against the front-line antimicrobials, chloramphenicol and trimethoprim/sulfamethoxazole but also against clinically important antimicrobial agents, such as β-lactams and fluoroquinolones, is also an emerging problem (Lunguya et al., 2013).

The spread of the antibiotic resistant potential in *Salmonella* is mainly attributed to integrons. Integrons are DNA elements, capable of capturing antimicrobial resistant genes and disseminating them using a mobile genetic element (MGE) such as a plasmid among bacteria. The class I integron is the most common integron type identified in multidrug-resistant (MDR) *Salmonella* and plays an important role in the dissemination of resistance genes among pathogens (Wright, 2010).

In developed countries, many surveys have been conducted at the molecular level to monitor the incidence of antibiotic-resistant *Salmonella* in animal farms (Melendez et al., 2010; Graciela et al., 2016). However, the extent of antibiotic-resistant *Salmonella* in many developing countries and the molecular mechanisms underlying this resistance remain unclear. Therefore, we selected large-scale animal farms as sample sites, collected swab samples, isolated *Salmonella* and characterized the molecular mechanisms of antimicrobial resistance.

## Materials and methods

### Samples and *Salmonella* isolation

From April to July 2016, rectal swabs were collected from healthy animals on farms in Qingdao, Jinan and Zibo regions in Shandong Province, China. All of the sampling sites were visited only once. In total, 1,105 samples were collected in a random manner from chickens (*n* = 325), ducks (*n* = 600), and pigs (*n* = 180). The samples were independently collected from individual animals, and the sample collection conformed to the cluster random sampling principle. Farms were chosen based on their scale with the following requirements: for chickens, the breeding stock was >150,000 heads; for ducks, the breeding stock was >100,000 heads, and for pigs, the breeding stock was >1,000 heads. The owners of each farm gave permission for rectal swab samples to be collected. The animals from which samples were extracted remained alive and did not undergo any surgery. Therefore, ethical approval was not required for the study because the sampling process did not harm the animals. All of the collected samples were transported in an ice box to our laboratory within 6 h for further bacteriological analysis.

Isolation and identification of *Salmonella* were performed as described previously (Yan et al., 2010), with some modifications. Briefly, swabbing samples were placed into a sterile plastic bag containing 100 ml of buffered peptone water (BPW) and mixed vigorously for 3 min. The BPW mixture was then incubated for 24 h at 37°C for pre-enrichment. Approximately 1 ml of pre-enrichment cultures were incubated in 10 ml of selenite cysteine (SC) broth and 10 ml of rappaport-vassiliadis (RV) broth at 42°C for 24 h, respectively. After selective enrichment, a loop-full of SC and RV broth cultures were streaked onto xylose lysine tergitol 4 (XLT4) agar and incubated at 37°C overnight. A minimum of two presumptive *Salmonella* colonies was confirmed by PCR using a previously described method (Malorny et al., 2003).

### *Salmonella* serotyping

According to the manufacturer's instructions, the serogroup and serovars of *Salmonella* isolates were determined according to the Kauffmann-White scheme by slide agglutination with O and H antigens (Tianrun Bio-Pharmaceutical, Ningbo, China).

### Antimicrobial susceptibility testing

A minimal inhibition concentration (MIC) assay, as described by the Clinical and Laboratory Standard Institute (Clinical Laboratory Standards Institute, 2013), was used in this study to test the susceptibility of 12 commonly used antibiotics (Table 1), including ampicillin (AMP), amikacin (AMK), enrofloxacin (ENO), ciprofloxacin (CIP), nalidixic acid (NA), florfenicol (FFN), tetracycline (TET), ceftiofur (CEF), gentamicin (GEN), neomycin (NEO), levofloxacin (LVX), and fosfomycin (FOS). *Escherichia coli* (ATCC 25922) and *Klebsiella pneumoniae* (ATCC 700603) were used as the quality control strains in this study. *Salmonella* isolates resistant to more than three classes of antimicrobials were defined as MDR isolates.

**Table 1:** Antimicrobials and the range of concentrations tested.

**Antimicrobials**	**Abbreviation**	**Concentration range (μg/mL)**
Ampicillin	AMP	0.06~256
Amikacin	AMK	0.5~512
Enrofloxacin	ENO	0.06~512
Ciprofloxacin	CIP	0.015~512
Nalidixic acid	NA	0.06~512
Florfenicol	FFN	0.5~512
Tetracycline	TET	0.5~512
Ceftiofur	CEF	0.06~512
Gentamicin	GEN	0.5~512
Neomycin	NEO	0.5~512
Levofloxacin	LVX	0.06~512
Fosfomycin	FOS	1~2.048

### Detection of class I integrons and antimicrobial resistance genes

Bacterial DNA was extracted using a TIANamp Bacteria DNA Kit (Tiangen, Beijing, China) according to the manufacturer's instructions. Conserved primers were used for the detection and identification of class I integrons using previously described primers and procedures (Kerrnet et al., 2002). PCR screening for β-lactamase-encoding genes *bla*_TEM_, *bla*_PSE−1_, *bla*_SHV_, and *bla*_CTX−M_ was performed as previously described (Li et al., 2013). Furthermore, PCR amplification was used to screen for plasmid-mediated quinolone resistance genes, *qnrA, qnrB, qnrC, qnrD*, and *qnrS*, which were the most frequently observed in China, using previously described primers (Ahmed et al., 2013). Finally, the florfenicol resistance gene, *floR*, was detected using previously described primers (Ahmed et al., 2013). The PCR products were purified and subsequently sequenced (Invitrogen, Beijing, China). The obtained DNA sequences were compared with those in GenBank using Basic Local Alignment Search Tool (BLAST).

### MLST

Seven housekeeping genes (*aroC, dnaN, hemD, hisD, purE, sucA*, and *thrA*) were used to characterize *Salmonella* by MLST. MLST was performed as described online (http://mlst.warwick.ac.uk/mlst/dbs/Senterica/documents/primersEnterica_html). All polymerase chain reaction products were purified and sequenced (Invitrogen, Beijing, China), and the alleles and STs were assigned according to the MLST scheme at http://mlst.warwick.ac.uk/mlst/dbs/Senterica.

### Data analysis

The statistical package SPSS (version 15.0, SPSS, Chicago, IL, USA) was used to compare the prevalence and MDR resistance rate of *Salmonella* isolated from chickens, ducks and pigs, and a *P*-value less than 0.05 was considered significant.

## Results

### Prevalence and serotypes of *Salmonella*

In this study, a total of 154 non-duplicate *Salmonella* isolates (154/1105, 13.9%) were recovered. From chickens, 78 *Salmonella* isolates were recovered (78/325, 24.0%) (Table 2), which was significantly higher than the *Salmonella* isolated from ducks and pigs (*P* < 0.05). Seventy-eight *Salmonella* isolates were divided into six serovars. The most common serovar was *Salmonella* enteritidis (69/78, 88.5%) (Table 3).

**Table 2:** Prevalence of *Salmonella* isolates from farm animals.

	**Chicken**		**Duck**		**Pig**	
**Locations**	**No. of samples**	**No. of posotive samples (%)**	**No. of samples**	**No. of posotive samples (%)**	**No. of samples**	**No. of posotive samples (%)**
Qingdao	100	17 (17%)	200	22 (11.0%)	60	7 (11.7%)
Jinan	115	34 (29.6%)	200	19 (9.5%)	60	8 (13.3%)
Zibo	110	27 (24.5%)	200	15 (7.5%)	60	5 (8.3%)
Total	325	78 (24.0%)	600	56 (9.3%)	180	20 (11.1%)

**Table 3:** Resistance phenotype, ST, incidence of class I integron, and resistance gens in *Salmonella* isolated from animals in farms.

**No**.	**Location**	**Farms**	**Serovar**	**ST**	**Resistance phenotype**	**Integrons/resistance genes**
1	Qingdao	Chicken	*S*. Enteritidis	11	AMP, CEF, ENO, TET	*bla*_TEM−1_,*qnrA*
2	Qingdao	Chicken	*S*. Enteritidis	11	AMP, TET	*qnrA*
3	Qingdao	Chicken	*S*. Enteritidis	11	AMP, CEF, NA, NEO, TET	Class I (*drfA1*-*aadA1*), *bla*_TEM−1_,*qnrA, qnrS*
4	Qingdao	Chicken	*S*. Enteritidis	11	AMP, ENO, NA, TET	*bla*_TEM−1_, *qnrA, qnrB*
5	Qingdao	Chicken	*S*. Enteritidis	11	AMP, CEF, CIP, ENO, GEN, NA, NEO	Class I (*aadA2*), *bla*_PSE−1_, *qnrB, qnrS*
6	Qingdao	Chicken	*S*. Enteritidis	11	AMP, CIP, ENO, FFN, GEN, NA, NEO	*bla*_TEM−1_, *bla*_CTX−M−55_, *qnrB, floR*
7	Qingdao	Chicken	*S*. Enteritidis	11	AMP, TET	*bla*_TEM−1_
8	Qingdao	Chicken	*S*. Enteritidis	11	AMP, ENO, NA, TET	*bla*_CTX−M−55_, *qnrB*
9	Qingdao	Chicken	*S*. Enteritidis	11	AMP, CEF, CIP, ENO, NA, TET	*qnrA, qnrB*
10	Qingdao	Chicken	*S*. Enteritidis	11	AMP, CIP, TET	*bla*_TEM−1_,*qnrA, qnrB*
11	Qingdao	Chicken	*S*. Indiana	17	AMP, CEF, CIP, ENO, FFN, NA, TET	Class I (*aadA2*), *bla*_TEM−1_, *qnrA, qnrB, qnrS, floR*
12	Qingdao	Chicken	*S*. Enteritidis	11	AMP, CEF, ENO, GEN, NA, TET	*bla*_PSE−1_, *qnrA, qnrS*
13	Qingdao	Chicken	*S*. Enteritidis	11		
14	Qingdao	Chicken	*S*. Enteritidis	11	AMP, CIP, TET	*qnrA*
15	Qingdao	Chicken	*S*. Enteritidis	11	AMP, NA	*qnrA*
16	Qingdao	Chicken	*S*. Enteritidis	11	AMP, ENO, NA, TET	*bla*_TEM−1_, *qnrA, qnrB*
17	Qingdao	Chicken	*S*. Enteritidis	11	NA	
18	Jinan	Chicken	*S*. Enteritidis	11	AMP, CIP, TET	*qnrA*
19	Jinan	Chicken	*S*. Thompson	26	AMP, CEF, GEN, NA, TET	*qnrA, qnrB*
20	Jinan	Chicken	*S*. Enteritidis	11	AMP, CIP, ENO, FFN, GEN, NA, NEO	Class I (*aadA2*), *bla*_TEM−1_, *qnrA, qnrB, floR*
21	Jinan	Chicken	*S*. Enteritidis	11	AMP, CEF, CIP, NA, TET	*bla*_TEM−1_, *qnrA, qnrB*
22	Jinan	Chicken	*S*. Enteritidis	11	AMP, ENO, NA, TET	*qnrA*
23	Jinan	Chicken	*S*. Enteritidis	11	AMP, TET	
24	Jinan	Chicken	*S*. Enteritidis	11	AMP, CIP, ENO, FFN, GEN, NA, NEO	Class I (*aadA2*), *bla*_TEM−1_, *qnrA, qnrB, floR*
25	Jinan	Chicken	*S*. Enteritidis	11	AMP, CEF, FFN, ENO, NA, TET	Class I (*aadA2*), *qnrA, qnrB, floR*
26	Jinan	Chicken	*S*. Thompson	26	AMP, ENO, NA, NEO, TET	*bla*_TEM−1_, *qnrA, qnrB*
27	Jinan	Chicken	*S*. Enteritidis	11	AMP, CEF, FFN, NEO, NA, TET	Class I (*drfA1*-*aadA1*), *bla*_PSE−1_, *qnrA, floR*
28	Jinan	Chicken	*S*. Enteritidis	11	AMP, CEF, NA, NEO, TET	*qnrA, qnrB*
29	Jinan	Chicken	*S*. Enteritidis	11		
30	Jinan	Chicken	*S*. Enteritidis	11	AMP, CEF, FFN, NEO, NA, TET	Class I (*aadA1*), *bla*_TEM−1_, *qnrA, floR*
31	Jinan	Chicken	*S*. Enteritidis	11	AMP, CEF, CIP, ENO, NA, TET	*qnrA, qnrB*
32	Jinan	Chicken	*S*. Enteritidis	11		
33	Jinan	Chicken	*S*. Enteritidis	11	AMP, GEM, NA, TET	*qnrA*
34	Jinan	Chicken	*S*. Typhimurium	19	AMP, CEF, CIP, FFN, NA, TET	*qnrB, floR*
35	Jinan	Chicken	*S*. Enteritidis	11	AMP, CEF, ENO, TET	*qnrA*
36	Jinan	Chicken	*S*. Enteritidis	11	AMP, CEF, CIP, FFN, NA, TET	Class I (*drfA17*-*aadA5*), *bla*_PSE−1_, *qnrA, qnrB, floR*
37	Jinan	Chicken	*S*. Enteritidis	11	AMP, CIP, TET	*qnrA, qnrB*
38	Jinan	Chicken	*S*. Enteritidis	11	AMP, CEF, CIP, ENO, FFN, GEN, NA	Class I (*drfA17*-*aadA5*), *bla*_TEM−1_, *bla*_PSE−1_, *qnrA, qnrB, floR*
39	Jinan	Chicken	*S*. Enteritidis	11	TET	
40	Jinan	Chicken	*S*. Enteritidis	11	AMP, ENO, NA, TET	*qnrA, qnrB*
41	Jinan	Chicken	*S*. Enteritidis	11	AMP, CIP, TET	*bla*_TEM−1_, *qnrA, qnrB, floR*
42	Jinan	Chicken	*S*. Enteritidis	11	AMP, CIP, TET	*qnrA, qnrB*
43	Jinan	Chicken	*S*. Enteritidis	11	AMP, CEF, FFN, NA, TET	*qnrA, qnrB, floR*
44	Jinan	Chicken	*S*. Enteritidis	11	AMP, ENO, NA, TET	*qnrB*
45	Jinan	Chicken	*S*. Enteritidis	11	NA	*qnrB*
46	Jinan	Chicken	*S*. Enteritidis	11	AMP, ENO, NA, TET	*bla*_PSE−1_, *qnrA, qnrB*
47	Jinan	Chicken	*S*. Enteritidis	11	AMP, CIP, TET	*qnrA*
48	Jinan	Chicken	*S*. Enteritidis	11		
49	Jinan	Chicken	*S*. Enteritidis	11	AMP, CIP, TET	*qnrA, qnrB*
50	Jinan	Chicken	*S*. Enteritidis	11	AMP, ENO, NA, TET	*qnrA, qnrB*
51	Jinan	Chicken	*S*. Indiana	17	AMP, CEF, CIP, ENO, FFN, NA, TET	Class I (*drfA17*-*aadA5*), *qnrA, qnrB, floR*
52	Zibo	Chicken	*S*. Enteritidis	11	AMP, CEF, ENO, GEN, NA, TET	Class I (*aadA1*), *qnrA, qnrB, qnrS*
53	Zibo	Chicken	*S*. Agona	28	AMP, CEF, ENO, NA, TET	*bla*_TEM−1_, *qnrA, qnrB*
54	Zibo	Chicken	*S*. Enteritidis	11	AMP, CEF, ENO, FFN, GEN, NA, TET	Class I (*drfA1*-*aadA1*), *bla*_TEM−1_, *bla*_CTX−M−55_, *qnrA, qnrS, floR*
55	Zibo	Chicken	*S*. Senftenberg	14	AMP, ENO, NA, TET	*qnrA, qnrB*
56	Zibo	Chicken	*S*. Enteritidis	11	AMP, CEF, FFN, NEO, NA, TET	Class I (*drfA1*-*aadA1*), *qnrB, floR*
57	Zibo	Chicken	*S*. Enteritidis	11	AMP, CIP, TET	*bla*_CTX−M−55_, *qnrA*
58	Zibo	Chicken	*S*. Enteritidis	11	AMP, CEF, ENO, NEO, NA, TET	*bla*_TEM−1_, *qnrA, floR*
59	Zibo	Chicken	*S*. Enteritidis	11	AMP, CEF, FFN, GEN, NA, TET	*bla*_TEM−1_, *bla*_PSE−1_, *qnrA, floR*
60	Zibo	Chicken	*S*. Enteritidis	11	AMP, CIP, TET	*qnrA, qnrB*
61	Zibo	Chicken	*S*. Enteritidis	11	AMP, ENO, NA, TET	*qnrB*
62	Zibo	Chicken	*S*. Enteritidis	11		
63	Zibo	Chicken	*S*. Enteritidis	11	AMP, ENO, NA, TET	*qnrA*
64	Zibo	Chicken	*S*. Enteritidis	11	AMP, TET	
65	Zibo	Chicken	*S*. Enteritidis	11	AMP, ENO, NA, TET	*bla*_TEM−1_
66	Zibo	Chicken	*S*. Enteritidis	11	AMP, ENO, NA, TET	*qnrA*
67	Zibo	Chicken	*S*. Enteritidis	11	AMP, CEF,GEN, NA, TET	*bla*_TEM−1_, *qnrB, floR*
68	Zibo	Chicken	*S*. Enteritidis	11	AMP, CIP, TET	*qnrB*
69	Zibo	Chicken	*S*. Enteritidis	11	AMP, CEF, NA, NEO, TET	*qnrA, qnrB, floR*
70	Zibo	Chicken	*S*. Enteritidis	11		
71	Zibo	Chicken	*S*. Enteritidis	11	AMP, CIP, TET	
72	Zibo	Chicken	*S*. Enteritidis	11	AMP, NA	
73	Zibo	Chicken	*S*. Indiana	17	AMP, CEF, CIP, FFN, GEN, NA, TET	Class I (*drfA1*-*aadA1*), *bla*_TEM−1_, *qnrA, floR*
74	Zibo	Chicken	*S*. Enteritidis	11	AMP, CEF, CIP, ENO, GEN, NA, NEO	*bla*_TEM−1_, *qnrA, qnrB*
75	Zibo	Chicken	*S*. Indiana	17	AMP, CEF, CIP, ENO, FFN, GEN, NA	Class I (*drfA1*-*aadA1*), *bla*_TEM−1_, *qnrA, qnrS, floR*
76	Zibo	Chicken	*S*. Enteritidis	11	AMP, CEF, CIP, FFN, GEN, TET	*qnrA, qnrB, floR*
77	Zibo	Chicken	*S*. Enteritidis	11	AMP, CEF, CIP, FFN, GEN, TET	Class I (*drfA1*-*aadA1*), *bla*_TEM−1_, *qnrB, floR*
78	Zibo	Chicken	*S*. Enteritidis	11	AMP, GEN, NA, TET	*qnrA, qnrB*
79	Qingdao	Duck	*S*. Typhimurium	34	CIP, ENO, GEN, NA, NEO, TET	*qnrA, qnrB*
80	Qingdao	Duck	*S*. Typhimurium	34	CIP, NA, NEO, TET	*qnrA*
81	Qingdao	Duck	*S*. Typhimurium	19	AMP	*bla*_TEM−1_
82	Qingdao	Duck	*S*. Enteritidis	11	AMP, CEF, CIP, ENO, FFN, NEO, GEN, NA, TET	Class I (*drfA1*-*aadA1*), *bla*_TEM−1_, *qnrB, floR*
83	Qingdao	Duck	*S*. Enteritidis	11	AMP	*bla*_TEM−1_
84	Qingdao	Duck	*S*. Typhimurium	19	AMP, NA	*bla*_TEM−1_, *qnrA, qnrB*
85	Qingdao	Duck	*S*. Enteritidis	11	AMP, CEF, ENO, GEN, NA, NEO, TET	Class I (*drfA1*-*aadA1*), *bla*_TEM−1_, *bla*_CTX−M−55_, *qnrB*
86	Qingdao	Duck	*S*. Enteritidis	11	CEF, CIP, ENO, FFN, GEN, NA, TET	Class I (*aadA2*), *qnrA, qnrB*
87	Qingdao	Duck	*S*. Enteritidis	11	AMP, TET	*bla*_TEM−1_
88	Qingdao	Duck	*S*. Enteritidis	11		*bla*_TEM−1_
89	Qingdao	Duck	*S*. Typhimurium	34	CIP, ENO, GEN, NA, NEO, TET	*qnrA, qnrB*
90	Qingdao	Duck	*S*. Enteritidis	11	CEF, CIP, ENO, FFN, GEN, NA, TET	*qnrA, floR*
91	Qingdao	Duck	*S*. Enteritidis	11	AMP, CEF, CIP, ENO, FFN, NEO, GEN, NA, TET	*qnrA, qnrB, floR*
92	Qingdao	Duck	*S*. Typhimurium	34	CEF, CIP, ENO, GEN, NA, NEO, TET	Class I (*aadA2*), *qnrB*
93	Qingdao	Duck	*S*. Enteritidis	11	CEF, CIP, ENO, FFN, GEN, NA, NEO, TET	*qnrA*
94	Qingdao	Duck	*S*. Enteritidis	11	CEF, CIP, ENO, FFN, GEN, NA, TET	*qnrA, qnrB*
95	Qingdao	Duck	*S*. Enteritidis	11	AMP, CEF, CIP, GEN, NA, NEO, TET	*bla*_TEM−1_, *qnrA*
96	Qingdao	Duck	*S*. Enteritidis	11	CEF, CIP, ENO, NA, TET	*qnrA, qnrB*
97	Qingdao	Duck	*S*. Enteritidis	11	CEF, CIP, ENO, NA, NEO, TET	*qnrA*
98	Qingdao	Duck	*S*. Enteritidis	11	CIP, ENO, GEN, NA, TET	*qnrA, qnrB*
99	Qingdao	Duck	*S*. Enteritidis	11	CEF, CIP, ENO, FFN, GEN, NA, NEO, TET	*bla*_TEM−1_, *qnrA, floR*
100	Qingdao	Duck	*S*. Enteritidis	11	CEF, CIP, NA, TET	*qnrB*
101	Jinan	Duck	*S*. Typhimurium	19	CIP, ENO, FFN, GEN, NA, NEO, TET	*qnrA, floR*
102	Jinan	Duck	*S*. Enteritidis	11	AMP, CIP, ENO, FFN, NA, NEO, TET	*bla*_TEM−1_, *qnrB*
103	Jinan	Duck	*S*. Typhimurium	19	AMP, CEF, CIP, NA, NEO, TET	*qnrA*
104	Jinan	Duck	*S*. Typhimurium	19	CIP, NA, NEO, TET	*qnrA, qnrB*
105	Jinan	Duck	*S*. Typhimurium	19	AMP, CEF, ENO, GEN, NA, NEO, TET	Class I (*aadA2*), *bla*_TEM−1_, *qnrB*
106	Jinan	Duck	*S*. Typhimurium	19	CIP, ENO, GEN, NA, TET	*qnrA, qnrB*
107	Jinan	Duck	*S*. Enteritidis	11	CEF, CIP, ENO, FFN, GEN, NA, NEO, TET	*qnrA, qnrB*
108	Jinan	Duck	*S*. Enteritidis	11	AMP, CEF, CIP, ENO, FFN, GEN, NA, TET	*bla*_TEM−1_, *qnrA, floR*
109	Jinan	Duck	*S*. Enteritidis	11	CIP, ENO, FFN, GEN, NA, TET	*qnrA, qnrB*
110	Jinan	Duck	*S*. Enteritidis	11	CEF, CIP, ENO, FFN, GEN, NA, TET	*qnrA, qnrB, floR*
111	Jinan	Duck	*S*. Enteritidis	11	AMP, CEF, CIP, ENO, NA, TET	*bla*_TEM−1_, *qnrB*
112	Jinan	Duck	*S*. Enteritidis	11	AMP, CEF, CIP, GEN, NA, NEO, TET	*bla*_TEM−1_, *bla*_PSE−1_, *qnrA*
113	Jinan	Duck	*S*. Enteritidis	11	CEF, CIP, ENO, FFN, GEN, NA, TET	*qnrA, qnrB, floR*
114	Jinan	Duck	*S*. Enteritidis	11	AMP, CEF, NA, NEO, TET	*bla*_TEM−1_, *qnrA, qnrB*
115	Jinan	Duck	*S*. Enteritidis	11	CEF, CIP, ENO, FFN, GEN, NA, NEO, TET	*qnrA, qnrB, floR*
116	Jinan	Duck	*S*. Enteritidis	11	CIP, ENO, GEN, NA, TET	*qnrA, qnrB*
117	Jinan	Duck	*S*. Enteritidis	11	CIP, GEN, NA, TET	*qnrA, qnrB*
118	Jinan	Duck	*S*. Enteritidis	11	AMP, CEF, CIP, NA, NEO, TET	Class I (*aadA2*), *bla*_TEM−1_, *qnrA, qnrB*
119	Jinan	Duck	*S*. Enteritidis	11	CIP, ENO, GEN, NA, TET	*qnrA, qnrB*
120	Zibo	Duck	*S*. Enteritidis	11	CEF, CIP, ENO, FFN, GEN, NA, TET	*qnrA, qnrB, floR*
121	Zibo	Duck	*S*. Enteritidis	11	AMP, CEF, CIP, ENO, FFN, NEO, GEN, NA, TET	Class I (*drfA1*-*aadA1*), *bla*_TEM−1_, *qnrA, qnrB, floR*
122	Zibo	Duck	*S*. Enteritidis	11	AMP, CEF, FFN, GEN, NA, NEO, TET	*qnrA, qnrB*
123	Zibo	Duck	*S*. Typhimurium	19	AMP, CEF, CIP, FFN, NA, NEO, TET	*qnrA, qnrB*
124	Zibo	Duck	*S*. Typhimurium	19	CEF, CIP, ENO, GEN, NA, TET	*qnrA, qnrB*
125	Zibo	Duck	*S*. Typhimurium	34	CIP, ENO, GEN, NA, NEO, TET	*qnrA, qnrB*
126	Zibo	Duck	*S*. Enteritidis	11	AMP, CEF, ENO, GEN, NA, NEO, TET	*bla*_TEM−1_, *bla*_PSE−1_, *qnrA, qnrB*
127	Zibo	Duck	*S*. Enteritidis	11	AMP, CEF, CIP, GEN, NA, NEO, TET	*qnrA, qnrB*
128	Zibo	Duck	*S*. Enteritidis	11	AMP, CEF, ENO, FFN, GEN, NA, NEO, TET	Class I (*drfA12*-*aadA2*), *bla*_TEM−1_, *qnrB, qnrS*
129	Zibo	Duck	*S*. Typhimurium	34	CEF, CIP, ENO, GEN, NA, TET	*qnrA*
130	Zibo	Duck	*S*. Typhimurium	34	AMP, CEF, CIP, NA, TET	*qnrA, qnrB*
131	Zibo	Duck	*S*. Enteritidis	11	AMP, CIP, ENO, FFN, GEN, NA, NEO, TET	*bla*_TEM−1_, *qnrA, qnrS, floR*
132	Zibo	Duck	*S*. Typhimurium	19	CIP, ENO, GEN, NA, NEO, TET	*qnrA, qnrB*
133	Zibo	Duck	*S*. Typhimurium	19	AMP, CEF, CIP, NA, TET	*qnrA, qnrB*
134	Zibo	Duck	*S*. Enteritidis	11	CEF, CIP, ENO, FFN, GEN, NA, TET	*qnrA, qnrB, floR*
135	Qingdao	Pig	*S*. Typhimurium	19	AMP, NA	*qnrA, qnrB, floR*
136	Qingdao	Pig	*S*. Typhimurium	19	AMP, TET	*bla*_TEM−1_, *qnrA, qnrB, floR*
137	Qingdao	Pig	*S*. Typhimurium	19		*qnrB, floR*
138	Qingdao	Pig	*S*. Typhimurium	34	AMP, CIP, ENO, FFN, LVX, NA, NEO, TET	*bla*_TEM−1_, *bla*_CTX−M−55_,*qnrA, qnrB, floR*
139	Qingdao	Pig	*S*. Derby	40	TET	*floR*
140	Qingdao	Pig	*S*. Derby	40	AMP	*bla*_TEM−1_,*qnrA, floR*
141	Qingdao	Pig	*S*. Typhimurium	19	AMP, CIP, FFN	*bla*_CTX−M−55_, *bla*_PSE−1_, *qnrA, qnrB, floR*
142	Jinan	Pig	*S*. Typhimurium	19	FFN, FOS, TET	*qnrA*,*qnrB, floR*
143	Jinan	Pig	*S*. Derby	40	AMP	*qnrA, floR*
144	Jinan	Pig	*S*. Derby	40		
145	Jinan	Pig	*S*. Typhimurium	19	AMP, CIP, FFN, LVX, NA, NEO, TET	Class I (*aadA2*), *bla*_TEM−1_,*bla*_PSE−1_, *qnrA, qnrB, floR*
146	Jinan	Pig	*S*. Typhimurium	34	AMP, CIP, FFN	*bla*_TEM−1_, *bla*_PSE−1_, *qnrA, qnrB, floR*
147	Jinan	Pig	*S*. Enteritidis	11	AMP, CEF, CIP, FFN, FOS, NA	*bla*_TEM−1_, *bla*_CTX−M−55_, *qnrA, qnrB, floR*
148	Jinan	Pig	*S*. Typhimurium	34	AMP, TET	*bla*_CTX−M−55_, *qnrA, qnrB, floR*
149	Jinan	Pig	*S*. Typhimurium	34		
150	Zibo	Pig	*S*. Enteritidis	11	AMP, CIP, FFN, NA	*bla*_TEM−1_, *bla*_PSE−1_, *qnrA, qnrB, floR*
151	Zibo	Pig	*S*. Typhimurium	34	AMP, CIP, ENO, FFN	*bla*_TEM−1_, *bla*_CTX−M−55_, *qnrA, qnrB, floR*
152	Zibo	Pig	*S*. Typhimurium	19	AMP, CIP, TET	*bla*_TEM−1_, *bla*_PSE−1_, *qnrA, qnrB, floR*
153	Zibo	Pig	*S*. Typhimurium	19	AMP, TET	*bla*_TEM−1_, *qnrA, qnrB, qnrS, floR*
154	Zibo	Pig	*S*. Enteritidis	3,007	AMP, TET	*bla*_CTX−M−55_, *qnrA, qnrB, floR*

From ducks, 56 *Salmonella* isolates were recovered (56/600, 9.3%) (Table 2), and they were divided into two serovars. The most common serovar was *Salmonella* enteritidis (38/56, 67.9%) (Table 3).

From pigs, 20 *Salmonella* isolates were recovered (20/180, 11.1%) (Table 2), and they were divided into three serovars. The most common serovar was *Salmonella* typhimurium (13/20, 65.0%) (Table 3).

### Antimicrobial susceptibility testing

Among 78 isolates from chickens, they were susceptible to amikacin, levofloxacin and fosfomycin. Most isolates were resistant to ampicillin (69/78, 88.5%) and tetracycline (61/78, 78.2%). In addition, 63 isolates (63/78, 80.8%) exhibited MDR (Table 3).

Among 56 isolates from ducks, they were susceptible to amikacin, levofloxacin and fosfomycin. Most isolates were resistant to tetracycline (52/56, 92.9%) and ciprofloxacin (45/56, 80.4%). In addition, 50 isolates (50/56, 89.3%) exhibited MDR (Table 3), which was significantly higher than the *Salmonella* isolated from chickens and pigs (*P* < 0.05).

Among 20 isolates from pigs, they were susceptible to amikacin and levofloxacin. Most isolates were resistant to ampicillin (15/20, 75.0%) and tetracycline (9/20, 45.0%). In addition, 9 isolates (9/20, 45.0%) exhibited MDR (Table 3).

### Characteristics of class I integrons and antimicrobial resistance genes

Among the 78 isolates recovered from chickens, 17 isolates (17/78, 21.8%) contained four groups of resistance gene cassettes, consisting of *drfA1-aadA1* (1.7 kb, *n* = 7), *aadA2* (1.2 kb, *n* = 5), *drfA17-aadA5* (2 kb, *n* = 3), and *aadA1* (1.2 kb, *n* = 2). Three β-lactamase genes were detected among the isolates, and *bla*_TEM−1_ (*n* = 25) was the most commonly isolated β-lactamase gene, followed by *bla*_PSE−1_ (*n* = 7) and *bla*_CTX−M−55_ (*n* = 4). Three plasmid-mediated quinolone resistance genes were detected among the isolates. *qnrA* (*n* = 53) was the most commonly isolated plasmid-mediated quinolone resistance gene, followed by *qnrB* (*n* = 44) and *qnrS* (*n* = 7). In addition, 23 isolates carried *floR* (Table 3).

Among the 56 isolates recovered from ducks, eight isolates (8/56, 14.3%) contained three groups of resistance gene cassettes, consisting of *aadA2* (1.2 kb, *n* = 4), *drfA1-aadA1* (1.7 kb, *n* = 3), and *drfA12-aadA2* (2 kb, *n* = 1). Three β-lactamase genes were detected among the isolates. *bla*_TEM−1_ was the most commonly isolated β-lactamase gene (*n* = 20), followed by *bla*_PSE−1_(*n* = 2) and *bla*_CTX−M−55_ (*n* = 1). Three plasmid-mediated quinolone resistance genes were detected among the isolates. *qnrA* was the most commonly isolated plasmid-mediated quinolone resistance gene (*n* = 44), followed by *qnrB* (*n* = 40) and *qnrS* (*n* = 2). In addition, 13 isolates carried *floR* (Table 3).

Among the 20 isolates recovered from pigs, one isolate (1/20, 5.0%) contained one group of a resistance gene cassette, consisting of *aadA2* (1.2 kb, *n* = 1). Three β-lactamase genes were detected among the isolates. *bla*_TEM−1_ was the most commonly isolated β-lactamase gene (*n* = 10), followed by *bla*_CTX−M−55_ (*n* = 6) and *bla*_PSE−1_ (*n* = 5). Three plasmid-mediated quinolone resistance genes were detected among the isolates. *qnrA* was the most commonly isolated plasmid-mediated quinolone resistance gene (*n* = 16), followed by *qnrB* (*n* = 15) and *qnrS* (*n* = 1). In addition, 18 isolates carried *floR* (Table 3).

### MLST

A total of nine STs among the 154 isolates were found. ST11 was the most common ST in both chickens and ducks, and it was represented by 69 and 38 *Salmonella* isolates, respectively. ST19 was the most common ST in pigs, and it was represented by eight *Salmonella* isolates (Table 3). The STs in this study were correlated with specific serovars, such as ST11 with *Salmonella* enteritidis, ST19 and ST34 with *Salmonella* typhimurium, and ST40 with *Salmonella* derby.

## Discussion

In this study, *Salmonella* spp. were recovered from chickens, ducks and pigs in Qingdao, Jinan and Zibo regions. For the chickens, the prevalence (24.0%) was significantly higher than that reported in Shanghai, China (4.5%) (Liu et al., 2010) but was lower than that reported from chicken farms in Egypt (41.0%) (Hanem et al., 2017). The prevalence (9.3%) in ducks was similar to that obtained from duck farms in Sichuan province (12.0%) (Li et al., 2013) but was lower than those reported in Penang, Malaysia (39.0%) (Adzitey et al., 2012), and in South Korea (65.2%) (Cha et al., 2013). For pigs, the occurrence ratio (11.1%) was similar to those reported in previous studies of *Salmonella* spp. in food products of animal origin in China (Jiang et al., 2006; Li et al., 2013) but was higher than that reported from conventional farms (3.5%) in Korea (Migma et al., 2015). Data on the prevalence of *Salmonella* in different studies were difficult to compare based on differences in regions, collection seasons, sample types, isolation methodologies, culture methods, culture media, and environmental conditions.

For serotyping, a total of seven serovars were found among the 154 isolates, including six from chickens, two from ducks, and three from pigs. The most common serotype in chickens and ducks was *Salmonella* enteritidis. This result was consistent with those from Shanxi province (Yang et al., 2010), but it was different from other reports that the dominant serotype in chicken farms was *Salmonella* Colindale in Chad (Tabo et al., 2013). The most common serotype in duck farms was *Salmonella* typhimurium (Martelli et al., 2016). The dominant serotype in pigs was *Salmonella* typhimurium, which was the most common serovar isolated from humans and it can lead to severe human and animal diseases (Deng et al., 2012), but it was different from other studies, where the dominant serotype in pig farms was *Salmonella* IIIb in Henan province (Kuang et al., 2015), and *Salmonella* derby in England and Wales (Miller et al., 2011). The difference in dominant serotype among animals may be due to differences in the pathogenicity of two serovars, geographical regions and diversities (Volf et al., 2010; European Centre for Disease Prevention Control, 2013).

Antimicrobial resistance in *Salmonella* is a threat to human public health. As shown in Table 3, the high rates of antimicrobial resistance were against tetracycline (72.0%) and ampicillin (69.4%) in all sources, which was similar to reports of *Salmonella* isolates from Africa, in which chickens exhibited resistance to tetracycline (93.0%) and ampicillin (47.0%) (Zishiri et al., 2016). These high resistance rates are due to its wide use in animal feed and were consistent with other reports (Piras et al., 2011; Shao, 2011; Bai et al., 2015). In addition, resistance to ciprofloxacin in 35.9% of chickens, 80.4% of ducks, and 30.0% of pigs deserves our attention because resistance to this antimicrobial agent may lead to the delay or failure of fluoroquinolone therapies (Van et al., 2007). In this study, all of the isolates were susceptible to amikacin, which may be because this antimicrobial is not used for therapeutic purposes in veterinary medicine or as a growth promoter in conventional animal fattening, and the result was consistent with other reports (Eva et al., 2015). In this study, MDR *Salmonella* isolates were frequently observed among chickens, ducks and pigs. In addition, MDR *Salmonella* is serotype-dependent (Clemente et al., 2014): the data provided evidence that *Salmonella* indiana, *Salmonella* typhimurium and *Salmonella* enteritidis were strongly associated with MDR phenotypes. Of particular concern, MDR strains could transfer to humans via animal or animal-derived products and pose a great risk to public health (Rosangela et al., 2016).

In this study, our results related to the incidence of class I integrons (26/154, 16.9%) were similar to the report in Sichuan (Li et al., 2013) but were higher than those reported in the USA, as class I integrons were identified in only 2.8% of the *Salmonella* isolates from bulk milk and milk filters (Van et al., 2013). In the present study, the incidence of class I integrons was significantly higher in *Salmonella* from chickens (21.8%) than *Salmonella* from pigs (5.0%). In addition, in this study, the *Salmonella* isolates carrying class I integrons included *Salmonella* enteritidis, typhimurium and indiana.

Production of β-lactamases is considered to be the main mechanism of resistance in Gram-negative bacteria to overcome penicillin-derived antibiotics, and the *bla*_TEM_ and bla_CTX−M_ ESBLs can hydrolyse third and fourth generation cephalosporins. In this study, a total of three β-lactamase genes were detected among the *Salmonella* isolates recovered from chickens, ducks and pigs: *bla*_TEM−1_, *bla*_PSE−1_, and *bla*_CTX−M−55_. Most of the isolates carried *bla*_TEM−1_, which was similar to the report in South Africa that *bla*_TEM−1_ was the most commonly identified β-lactamase gene in *Salmonella* isolates from food-producing animals (Igbinosa, 2015). In addition, in this study, most isolates carried *bla*_TEM−1_ and *bla*_CTX−M−55_, which confer resistance to ampicillin.

Quinolones are the first choice for the treatment of invasive and systemic salmonellosis that occurs in humans and animals (Dimitrov et al., 2007). A total of three quinolone resistance genes were detected among the *Salmonella* isolates recovered from chickens, ducks and pigs: *qnrA, qnrB* and *qnrS. qnrA* was the most commonly isolated plasmid-mediated quinolone resistance gene consistent with a report in Henan, where *qnrA, qnrB* and *qnrS* were identified in *Salmonella* strains isolated from retail food with an incidence of 46.6, 12.7, and 19.5%, respectively (Yang et al., 2013). It is well known that *qnr* genes confer only low-level resistance to fluoroquinolones, and accumulation of quinolone resistance-determining region (QRDR) mutations is necessary for *S*. enterica to be resistant to fluoroquinolone, especially ciprofloxacin (Eaves et al., 2004). In this study, most *Salmonella* isolates containing a plasmid-mediated quinolone resistance gene were resistant to ciprofloxacin, nalidixic acid and gentamicin.

Florfenicol, a new chemosynthesis broad spectrum antibiotic of chloramphenicol analogs, is a fluorinated derivative of thiamphenicol. It is not approved for human use. In this study, *floR* was identified in 35.1% of *Salmonella* strains isolated from chickens, ducks and pigs, which was significantly higher than that reported in Egypt (1.0%) (Ahmed and Shimamoto, 2012). In addition, *floR* was identified in 90.0% of *Salmonella* strains isolated from pigs in this study. In this study, most *Salmonella* isolates containing the *floR* gene were resistant to florfenicol.

MLST results reveal that a total of nine STs were identified in this study. ST11 was the most frequent genotype that was recovered in chickens and ducks, and ST19 was the most frequent genotype that was recovered in pigs. ST11 belongs to *Salmonella* enteritidis, and ST19 belongs to *Salmonella* typhimurium; they all have continually been reported to cause human salmonellosis in recent years (Cai et al., 2016; Kang et al., 2017). In addition, our results revealed that the MLST patterns were generally associated with serotypes and provided a reliable prediction of the *Salmonella* serovars, which was consistent with previous research (Achtman et al., 2012).

## Conclusion

The prevalence of *Salmonella* was higher in the animal farms. Moreover, many serovars reported in humans and MDR *Salmonella* were recovered in this study. The high rates of MDR *Salmonella*, class I integrons and antibiotic resistance gene positive isolates detected suggest that measures must be taken to facilitate the reasonable use of antimicrobials in animal husbandry. Therefore, continuous surveillance of *Salmonella* and associated antimicrobial resistance in *Salmonella* of animals is essential to detect emerging *Salmonella* serovars and associated resistance genes.

## Author contributions

SS, XZ, contributed to the conception of the study; WC, XZ; contributed significantly to analysis and manuscript preparation; XZ, performed the data analyses and wrote the manuscript; XZ, JY, BZ: helped perform the analysis with constructive discussions.

### Conflict of interest statement

The authors declare that the research was conducted in the absence of any commercial or financial relationships that could be construed as a potential conflict of interest.
